# A place for play? The influence of the home physical environment on children’s physical activity and sedentary behaviour

**DOI:** 10.1186/1479-5868-10-99

**Published:** 2013-08-17

**Authors:** Clover Maitland, Gareth Stratton, Sarah Foster, Rebecca Braham, Michael Rosenberg

**Affiliations:** 1 School of Sport Science, Exercise and Health, University of Western Australia, 35 Stirling Highway, Crawley, WA 6009, Australia; 2 Applied Sports Technology Exercise Medicine Research Centre, School of Engineering, Swansea University, Singleton Park, Swansea SA2 8PP, Wales; 3 Centre for Built Environment and Health, School of Population Health, University of Western Australia, 10 Stirling Highway, Crawley, WA 6009, Australia

**Keywords:** Home environment, Physical activity, Sedentary behaviour, Children, Adolescents, Review

## Abstract

The home environment is an important influence on the sedentary behaviour and physical activity of children, who have limited independent mobility and spend much of their time at home. This article reviews the current evidence regarding the influence of the home physical environment on the sedentary behaviour and physical activity of children aged 8–14 years. A literature search of peer reviewed articles published between 2005 and 2011 resulted in 38 observational studies (21 with activity outcomes, 23 with sedentary outcomes) and 11 experimental studies included in the review. The most commonly investigated behavioural outcomes were television watching and moderate to vigorous physical activity. Media equipment in the home and to a lesser extent the bedroom were positively associated with children’s sedentary behaviour. Physical activity equipment and the house and yard were not associated with physical activity, although environmental measures were exclusively self-reported. On the other hand, physical activity equipment was inversely associated with sedentary behaviours in half of studies. Observational studies that investigated the influence of the physical and social environment within the home space, found that the social environment, particularly the role of parents, was important. Experimental studies that changed the home physical environment by introducing a television limiting device successfully decreased television viewing, whereas the influence of introducing an active video game on activity outcomes was inconsistent. Results highlight that the home environment is an important influence on children’s sedentary behaviour and physical activity, about which much is still unknown. While changing or controlling the home physical environment shows promise for reducing screen based sedentary behaviour, further interventions are needed to understand the broader impact of these changes. Future studies should prioritise investigating the influence of the home physical environment, and its interaction with the social environment, on objectively measured sedentary time and home context specific behaviours, ideally including technologies that allow objective measures of the home space.

## Introduction

Changes to the environment in recent years have contributed to an increase in sedentary behaviour and a decline in activity [1]. The home environment is an important sphere of influence on the physical activity (PA) and sedentary behaviour of children. It is especially relevant for those who have limited independent mobility and spend much of their time at home and indoors [2,3], thereby potentially affecting PA participation and resultant health outcomes [4-6]. More recently, time spent sedentary, in particular watching television, has been associated with detrimental health effects including overweight and obesity, reduced fitness and poorer social and cognitive skills [7,8]. Still, many children do not meet health recommendations for PA and sedentary behaviour [9,10]. Thus, understanding the potential impact of the home environment on the sedentary and activity behaviours of children is vital for developing effective interventions.

Sedentary behaviour and PA are part of a movement continuum [11]. PA can be of light, moderate or vigorous intensity and at home may include unstructured play, exercise and chores [12]. Sedentary behaviours use low levels of energy (≤ 1.5 METs) while sitting or reclining, such as watching television, using a computer and reading, and are distinct from insufficient PA, also termed as inactivity [1]. Notably, reviews have concluded the independence of moderate to vigorous physical activity (MVPA) and sedentary behaviour in children [13-16]. Therefore while sedentary behaviour and PA coexist in the home space, they are distinct behaviours influenced by different factors [11].

Ecological models emphasise individual, social and physical environmental influences on PA and sedentary behaviour [13,17-19]. Consequently, a large body of literature exists on social environmental influences [20] and the built environment at the neighbourhood level [21,22], while the home physical environment has received less attention. Qualitative studies have identified lack of yard space and sedentary entertainment options, such as televisions and computers, as barriers to children’s PA, especially active play [23-26]. These factors, along with home design, have also been noted as influences on electronic media use [27]. However, previous reviews of correlates have not located any studies that have investigated the home physical environment with the exception of PA and media equipment [13,14,16,28,29], or considered interactions between physical and social environmental influences within the home space.

Across the world, home environments are rapidly changing. House sizes in countries such as Australia and the USA have increased, while block and yard sizes have decreased [30]. In contrast lack of indoor and outdoor space is a concern in the UK [31,32]. Additionally, new electronic media technologies such as wireless broadband, multifunctional devices and interactive video games are now an integral part of homes in developed countries. Time use studies have found that leisure time at home indoors is more likely to be sedentary, while time at home in the garden is more likely to be active [33,34]. Accordingly, there is a potentially important link between location within the home space and children’s PA and sedentary behaviours.

Thus, it is timely to review the influence of the home physical environment on children’s PA and sedentary behaviour. The aims of this review were to: (1) examine the impact of interventions that change the home physical environment on children’s PA and sedentary behaviours; (2) summarise the association between home physical environmental factors and children’s PA and sedentary behaviours; (3) explore the relationship of physical and social environmental factors operating within the home space; and (4) highlight current evidence limitations, measurement issues and future research directions. The time of transition from childhood to adolescence, known as preadolescence, represents a specific stage [35] and has been chosen as PA levels decrease [36,37] and sedentary screen based behaviour is high [13,38]. The review commences in 2005 to reflect recent changes in homes and build upon evidence from previous reviews of PA and sedentary behaviour by van der Horst et al. [14] and others [28,29].

## Methods

### Search procedure

**Figure 1:**
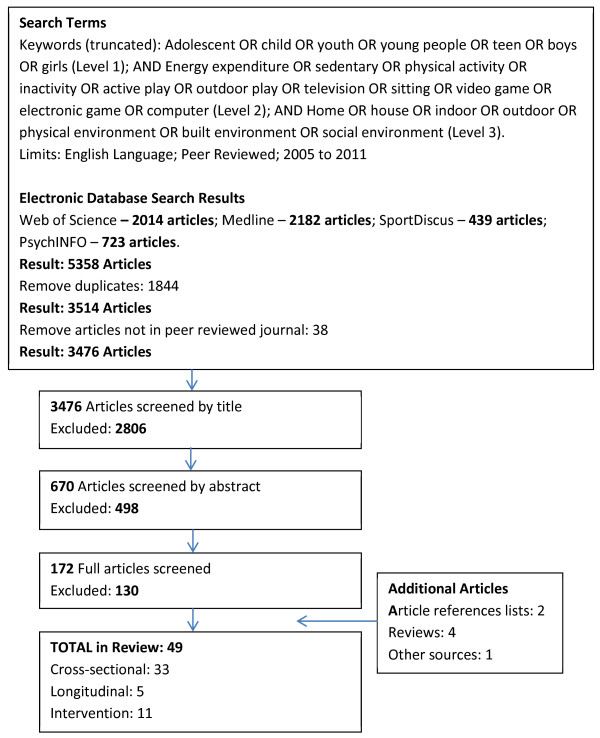
Literature search flow chart.

Medline, Web of Science, PsychInfo and Sportdiscus databases were searched for quantitative studies examining the relationship between the physical home environment and preadolescent children’s sedentary behaviours and PA. Combinations of key words were entered in three levels: children; activity; and home environment (Figure 1). The search was limited to English language peer reviewed journal articles published between 2005 and 2011.

### Inclusion/Exclusion criteria

Inclusion criteria were: (1) sample of healthy children with mean age of between eight and 14 years (at baseline); and (2) outcome variable of sedentary behaviour or PA, including overall time spent sedentary or in PA, or time spent in specific behaviours that potentially occur within the home, such as television watching, video game play, active play and leisure time PA. While preadolescence has most commonly been defined as nine to 13 years [35], some studies have also included those aged eight and 14 years within this group [39]. Therefore, this age range was chosen to ensure all appropriate studies were included. Where studies included multiple age groups only the results from groups with a mean age of eight to 14 years were included in the review. Studies with outcomes of vigorous PA only, school based PA, active transport and structured sport were excluded. Observational studies were required to: (1) include at least one home physical environmental factor as an independent variable, for example home equipment or yard size; and (2) examine an association between the independent and outcome variable. Studies that included only neighbourhood level built environmental variables or home social environmental variables were excluded. Studies with combined independent measures, such as home and neighbourhood facilities, were also excluded. Experimental studies were included if they: (1) contained at least one strategy that changed the home physical environment, such as adding or removing equipment; and (2) reported changes from baseline in the outcome variable.

### Selection process

Articles were screened in three phases. One reviewer read the title, then abstract and finally the full text, eliminating articles that did not meet the inclusion criteria at each screening phase. Twenty per cent of articles remaining at abstract level were independently screened by a second reviewer to confirm eligibility. Where ambiguity remained, a conclusion was reached by discussion between reviewers. Reference lists from selected articles and relevant review papers were searched for additional articles meeting inclusion criteria.

### Quality assessment

To identify the best available evidence and provide a guide to quality, each paper was assessed according to National Institute for Health and Clinical Excellence (NICE) quality appraisal checklists [40]. Each article received an overall score for internal and external validity, with ‘-’ representing the lowest validity (few criteria fulfilled); ‘+’ representing moderate validity (some criteria fulfilled); and ‘++’ representing the highest validity (all or most criteria fulfilled).

### Data analysis

Evidence tables summarising the study population, independent and outcome variables, analysis, results, quality assessment and intervention (where applicable), were constructed separately for observational and intervention studies according to NICE methods [40]. To summarise observational studies, all home physical environmental variables were categorised into: house and yard, PA equipment, media equipment, and bedroom media equipment. Home social environmental variables were categorised into: family rules, family social support (encouragement and co-participation/viewing) and family behaviour (sedentary behaviour and PA). As papers included a wide variety of sedentary behaviour and PA outcome variables, studies were designated as having either a sedentary or PA outcome and results were analysed in these two groups. Studies investigating both outcomes were included in both groups. Positive (+), negative (−) and null (0) associations significant at p<0.05 from the highest level of multivariate analysis were extracted and are presented in Tables 1, 2 and 3, unless noted. Where studies analysed data by gender these results are reported separately in the Tables. For studies including analysis of multiple groups based on other criteria (e.g. age or country groups), time specific outcomes (e.g. weekend vs weekday screen time) or reporting methods (e.g. parent and self-report), at least half of the analyses must have shown an association in the reported direction. Results of studies were synthesised by totalling the number of studies reporting an association in a given direction. These totals are reported in the written results to provide overall trends for each of the key home environmental variables.

**Table 1:** Summary of observational studies with sedentary behaviour outcomes only

**Author (Year); Country**	**Sample characteristics (Number, Age, % Male)**	**Outcome variables**	**Home physical and social environment independent variables**							**Adjustments**	**Internal validity**	**External validity**
			**House & yard**	**PA equipment**	**Media equipment**	**BR media equipment**	**Family rules**	**Family support**	**Family behaviour**			
**Sedentary Outcome Only - Cross-Sectional Studies**												
Cui (2011); China [41]	n=986; 6–18 yrs; 53%	**Screen time** (SR,PR)			**+**	**+**	**0**	**+**		Age, income, sex, residence, clustering	+	+
Devis-Devis (2009); Spain^E^[42]	n=323; 12–16 yrs; 46%	**TV time;**			**0**					Not noted	+	+
		**e-game time;**			**+**							
		**Mobile ph time** (SR) (V,R)			**+**							
Granich (2011); Aust. [43]	n=298; 11–12 yrs; 49%	**Screen time** (SR) (R)			**0**	**+**	**+/-**	**+**	**0**	Not included (NS) except for SES (school day), gender (weekend day); analysed by school/weekend day	+	+
Hardy (2006); Aust. [44]	n=343; 12–13 yrs; 50%	**TV time** (SR) (R)			**+**	**0**	**0**	**+**	**+**	Not included (NS)	+	++
Hesketh (2007); Aust.^A B D^[45]	n=895; grade 5 & 6; 46%	**TV Time** (PR) **(**R)			**+**	**+**	**-**	**+**		School clustering	+	+
Hoyos Cillero (2011); Spain [46]	n=503; 10–13 yrs; ~50%	**TV time;**			**+F,0M**	**0F,0M**	**0F,-M**		**0F,+M**	Child & parent BMI, parent ed.; school clustering; stratified by age, gender; analysed by week/weekend day	+	+
		**Screen time** (SR) (R)			**0F,0M**	**0F,0M**	**0F,0M**		**0F,+M**			
Hume (2010); The Netherlands [47]	n=338; 12–15 yrs; 55%	**TV time** (SR)		**0**		**0**	**0**		**+**	School clustering	+	+
Jago (2008); Europe [48]	n=2670; grade 3 & 9; 49%	**TV time;**			**0**	**0**				Grade, gender, father & mother income, obesity; school clustering, country (overall); stratified by country	+	++
		**e-game time** (SR)			**0**	**0**						
Norman (2005); USA [49]	n=878; 11–15 yrs; 46%	**Sedentary time** (SR) (R)		**0F,0M**			**-F, 0M**	**+F,0M**		Age, ethnicity, BMI, education; stratified by gender	+	+
Patriarca (2009); Italy [50]	n=987; 11–16 yrs; 50%	**TV Time;**			**0**	**0**	**0**			Age, gender, no. of siblings, both parents in household, parent working activity, sport activity	+	++
		**Computer time;**			**+**	**+**	**0**					
		**e-game time** (SR)			**0**	**0**	**-**					
Ramirez (2011); USA [51]	n=160; mean 14.6 yrs; 48%	**TV time;**				**+**	**-**			Not noted; analysed by SR/PR	+	+
		**e-game time;**				**+**	**-**					
		**Computer time** (SR,PR) (R)				**0**	**-**					
Springer (2010); USA [52]	n=734; grade 4; 50%	**TV time** (SR)				**0**	**-**		**+**	Gender, ethnicity, age, language, data collection period, school clustering; analysed by week/weekend day	+	+
van Sluijs (2010); Europe [53]	n=2107; 9–10 & 14–15 yrs; 46 & 56%	**Sedentary time** (Acc) (V)			**0**	**0**			**0**	Grade, sex; stratified by country	++	++
van Zutphen (2007); Aust. [54]	n=1926; 4–12 yrs; 49%	**TV time** (PR)			**+**	**+**	**-**			Parent ed., income, SES, no. of adults & children	+	+
Zabinski (2007); USA^C^[55]	n=878; 11–15 yrs; 48%	**Sedentary time** (SR)		**-**			**-**	**0**		Not noted	+	+
**Sedentary Outcome Only - Longitudinal Studies**												
Te Velde (2011); The Netherlands^B^[56]	n=12654; 11–17 yrs; 54%	**TV time** (SR) (R)				**+**	**-**		**+**	Age, sex, school level (SES), ethnicity	++	++
Willoughby (2008); Canada [57]	n=1591; grade 9 & 10; 51%	**e-game use;**			**0**					Parent ed., baseline value of outcome, gender	+	+
		**Internet use** (SR)			**0**							

Longitudinal studies include age at baseline; E-games include electronic & computer games; Computer includes computer and internet use; All analysis at highest multivariate level unless otherwise noted; Significance at p<0.05 unless otherwise noted; M (male) & F (female) entered separately where stratified as such; For studies with multiple groups or time specific outcomes at least half of analysis must show an association in the given direction (‘+’ or ‘-‘) with an independent variable; Adjustments listed as stated in paper; *SR* self report, *PR* parent report, *Acc* accelerometer, *V* validity reported, *R* reliability reported, *NS* potential confounders not included in final analysis as found to be non-significant, *SES* socioeconomic status, *BMI* body mass index, *A* bivariate analysis, *B* mediation analysis, *C* cluster analysis, *D* significance p<0.1, *E* SEM analysis.

**Table 2:** Summary of observational studies with physical activity outcomes only

**Author (Year); Country**	**Sample characteristics (Number, Age, % Male)**	**Outcome variables**	**Home physical and social environment independent variables**							**Adjustments**	**Internal validity**	**External validity**
			**House & yard**	**PA equipment**	**Media equipment**	**BR media equipment**	**Family rules**	**Family support**	**Family behaviour**			
**Physical Activity Outcome Only - Cross-Sectional Studies**												
Aarts (2010); The Netherlands [58]	n=4297; 7–9 yrs, 10–12 yrs; M/F	**Outdoor Play** (PR)	**+F,+M**		**0F,0M**	**+F,+M**	**0F,0M**			Parent ed., school clustering; stratified by age, gender	+	+
Erwin (2007); USA ^A B D^[59]	n=47; 6–13 yrs; 70%	**PA level** (SR)		**0**	**0**					None	-	-
Haerens (2009); Belguim [60]	n=62; grade 7 & 8; 19%	**MVPA time** (Acc) (V,R)		**0**	**0**			**0**		Age, SES, gender	+	+
Kerr (2008); USA [61]	n=839; 11–15 yrs; 42%	**MVPA level** (SR) (V)		**+F,0M**					**0F,0M**	Age, ethnicity, parent ed., parent PA; stratified by gender	+	-
Li (2006); China [62]	n=1787; 11–17 yrs; 50%	**Inactivity** (SR)	**0F,0M**		**0F,0M**		**0F,0M**	**0F,-M**		Clustering; stratified by gender	+	++
Maddison (2009); NZ^C^[63]	n=110; 12–17 yrs; 57%	**MVPA time** (Acc);		**0**						Not noted	+	-
		**MVPA time** (SR) (V)		**+**								
McMinn (2011); England [64]	n=2071; 9–10 yrs; 48%	**PA counts** (Acc)	**0**		**0**			**+**		Sex, age quartiles, mth of measurement, ethnicity, school clustering	++	++
Page (2010); UK [65]	n=1300; 10–11 yrs; 50%	**Outdoor play** (SR)	**0F,0M**							Daylight, neighbourhood deprivation, pubertal stage, BMI; stratified by gender	+	+
Ridgers (2010); UK [66]	n=110; 9–10 yrs; 41%	**MVPA time** (Acc) (V,R)		**0**	**-**					School clustering; analysed by week/weekend day time	+	+
Spinks (2006); Aust. [67]	n=518; 5–12 yrs; 54%	**Inactivity** (PR)		**0**						School clustering, gender, age, maternal ed., school transport, organised activity, family size, TV/computer use	+	+
Trang (2009); Vietnam [68]	n=2660; 11–16 yrs; 50%	**Inactivity** (SR) (V)	**0F,-M**		**0F,0M**		**0F,0M**	**0F,0M**		Clustering; sample weighted; stratified by gender	++	++
Veitch (2010); Aust. [69]	n=187; 8–9 yrs; 53%	**Outdoor play** (PR) (R)	**0**					**0**		Not noted; analysed by week/weekend day	+	+
Wong (2010); Hong Kong [70]	n=29,139; mean 14.5/6 yrs; 44%	**MVPA time** (SR) (V,R)			**+/-F,-M**					School clustering, age, family affluence, parent ed.; stratified by sex	++	++
**Physical Activity Outcome Only – Longitudinal Studies**												
Crawford (2010); Aust. [71]	n=301; 10–12 yrs; 46%	**MVPA time** (Acc) (V)		**0F,0M**	**0F,0M**	**0F,0M**	**+F,0M**	**+F,0M**	**0F,+M**	School clustering, baseline age, average zBMI; analysed by sex	++	++
Wilson (2011); USA [72]	n=198; grade 6; 47.5%	**MVPA time** (Acc)		**0**			**0**	**+**		Baseline MVPA & BMI, free lunch, ethnicity, parent ed., gender, intervention, school clustering	++	++

Longitudinal studies include age at baseline; *MVPA* moderate to vigorous physical activity; Inactivity defined as insufficient MVPA; All analysis at highest multivariate level unless otherwise noted; Significance at p<0.05 unless otherwise noted; M (male) & F (female) entered separately where stratified as such; For studies with multiple groups or time specific outcomes at least half of analysis must show an association in the given direction (‘+’ or ‘-‘) with an independent variable; Adjustments listed as stated in paper; *SR* self report, *PR* parent report, *Acc* accelerometer, *V* validity reported, *R* reliability reported, *SES* socioeconomic status, *BMI* body mass index, *A* bivariate analysis, *B* significance p<0.01; *C* SEM analysis; *D* independent measure home environment summary including both physical activity and media equipment.

**Table 3:** Summary of observational studies with sedentary behaviour and physical activity outcomes

**Author (Year), Country**	**Sample characteristics (Number, Age, % Male)**	**Outcome variables**	**Home physical and social environment independent variables**							**Adjustments**	**Internal validity**	**External validity**
			**House & yard**	**PA equipment**	**Media equipment**	**BR media equipment**	**Family rules**	**Family support**	**Family behaviour**			
**Sedentary and Physical Activity Outcome - Cross-Sectional Studies**												
Hume (2005); Aust.^A B^[73]	n=127; 10 yrs, 52%	**Sedentary time;**		**0F,0M**	**0F,0M**	**0F,0M**				Not included (NS); stratified by sex	+	+
		**LPA;**		**0F,0M**	**0F,0M**	**0F,0M**						
		**MPA** (Acc) (V, R)		**0F,0M**	**0F,0M**	**0F,+M**						
Roemmich (2007); USA [74]	n=88; 8–12 yrs; 50%	**TV time** (SR & PR);			**+**					Age, SES, % overweight, Acc wear time (PA), gender	+	+
		**PA counts;**			**0**							
		**MVPA time** (Acc) (V)			**0**							
Rosenberg (2010); USA [75]	n=189; 12–18 yrs; 49% n=116; 5–11 yrs; 48%	**TV time;**		**-**	**+**	**+**				Age, gender, race/ethnicity, household income, no. of children; stratified by age; analysed by PR/SR	+	+
		**Sedentary time** (SR & PR);		**0**	**+**	**+**						
		**MVPA time** (SR & PR) (V)		**+**	**0**	**0**						
Salmon (2005); Aust. [76]	n=878; 10–12 yrs; M/F	**TV Time** (PR) (V,R);			**0F,0M**	**0F,0M**	**-F,-M**	**0F,+M**	**+F,+M**	Maternal ed., school clustering, weight (girls TV model); stratified by gender	++	++
		**PA counts (low)** (Acc) (V)			**0F,+/-M**	**0F,0M**	**-F,+M**	**+F,0M**	**+F,+M**			
Sirard (2010); USA^C^[77]	n=575; 10–17 yrs; 49%	**Sedentary time** (Acc) (V,R);		**-**	**+**	**0**				Gender, age, ethnicity, parent ed., pubertal status, people in home, parent BMI, mth of data collection, free lunch, study cohort, school clustering; stratified by gender (screen time)	+	+
		**Screen time** (SR);		**-F,0M**	**+F,0M**	**0F,0M**						
		**MVPA time** (Acc) (V)		**+**	**0**	**0**						
**Sedentary and Physical Activity Outcome - Longitudinal Studies**												
Delmas (2007); France [78]	n=379, 12 yrs; 51%	**TV time;**				**0F,+M**				School clustering, sexual maturity, SES; stratified by gender	++	+
		**Reading** (SR) (V,R);				**-F,-M**						
		**Free** &**Club PA** (SR) (V)				**0F,0M**						

Longitudinal studies include age at baseline; *PA* physical activity, *LPA* light physical activity, *MPA* moderate physical activity, *MVPA* moderate to vigorous physical activity; All analysis at highest multivariate level unless otherwise noted; Significance at p<0.05 unless otherwise noted; M (male) & F (female) entered separately where stratified as such; For studies with multiple groups or time specific outcomes at least half of analysis must show an association in the given direction (‘+’ or ‘-‘) with an independent variable; Adjustments listed as stated in paper; *SR* self report, *PR* parent report, *Acc* accelerometer, *V* validity reported, *R* reliability reported, *NS* potential confounders not included in final analysis as found to be non-significant, *SES* socioeconomic status, *BMI* body mass index, *A* bivariate analysis; *B* independent measures taken from map drawn by sample; *C* PA equipment measure also includes physical activity to media equipment ratio.

## Results

Thirty-eight observational studies and 11 experimental studies were included in the final review (Figure 1). Most studies were conducted in high income countries including the USA [49,51,52,55,59,61,72,74],[75,77,79-83], Australia [43-45,54,67,69,71,73,76], UK [64-66,84], New Zealand [63,85-87] and The Netherlands [47,56,58,88]. Other countries contributing studies included China [41,62], Spain [42,46], Canada [57,89], Italy [50], Belgium [60], Vietnam [68], Hong Kong [70] and France [78]. Two studies included multiple European countries [48,53].

### Observational studies

Thirty-eight observational studies were identified including 33 cross-sectional and 5 longitudinal studies. Due to the low number of longitudinal studies, observational studies were analysed as one group. However, where applicable, the results of longitudinal studies are also reported separately to differentiate this stronger class of evidence. Nine studies scored the highest internal validity [53,56,64,68,70-72,76,78] and twelve scored the highest external validity [44,48,50,53,56,62,64,68],[70-72,76].

Studies investigated a variety of outcomes with 17 measuring sedentary outcomes (Table 1), 15 measuring PA outcomes (Table 2), and six measuring both (Table 3). In studies with sedentary outcomes, the most common was TV time (15 studies), followed by sedentary time (6 studies), electronic game use (5 studies), screen time (4 studies), computer/internet use (3 studies), mobile phone use (1 study) and reading (1 study). Two studies used accelerometers only [53,73] and one study used accelerometers with self-report to capture sedentary outcomes [77]. The remainder of studies used either self-report (13 studies [42-44,46-50,52,55-57,78]), parent report (3 studies [45,54,76]) or both (4 studies [41,51,74,75]).

PA outcomes included MVPA (11 studies), outdoor/free play (4 studies), average activity level (3 studies), inactivity (3 studies), moderate PA (1 study) and light PA (1 study). Ten studies used accelerometers [60,63,64,66,71-74,76,77], nine used self-report [59,61-63,65,68,70,75,78] and four used parental report [58,67,69,75]. Two incorporated more than one data collection method [63,75].

#### The home physical environment

Measures of the home physical environment included media equipment in the home (25 studies), media equipment in the child’s bedroom (20 studies), PA equipment (14 studies) and the parameters of the house and yard (6 studies). Two studies employed a home environment summary including both PA and media equipment [59,77]. Home physical environmental measures were collected via self-report in 19 studies [42-44,47,48,50,52,53,56,57],[59,60,63-66,70,73,78], parental report in 14 studies [45,49,54,55,58,61,62,67-69],[71,72,76,77] and five studies included both [41,46,51,74,75]. One study used an inventory implemented by parents [77] and no studies used objective measures of the home physical environment. Five studies reported the validity and reliability of the home physical environmental measure, ten reported reliability only and two reported validity only.

Media equipment within the home was positively associated with children’s sedentary behaviours in 10 of 16 studies [41,42,44-46,50,54,74,75,77]. The one longitudinal study that investigated this relationship found no association between computers in the home and e-game or internet use [57]. For bedroom media equipment, nine of 18 studies found a positive association with sedentary behaviours [41,43,45,50,51,54,56,75],[78], including both longitudinal studies that investigated TV time [56,78]. One of these longitudinal studies also found an inverse relationship between a bedroom TV and reading [78]. There were limited and inconsistent associations between media equipment in the home and PA outcomes. Three of 14 studies found negative associations and two of these also found positive associations for different equipment measures [66,70,76]. Two of seven studies of bedroom media equipment found a positive association with PA [58,73]. The two longitudinal studies in this group showed no association between media equipment and PA [71,78].

PA equipment was positively associated with PA outcomes in four of eleven studies [61,63,75,77], with no association found in the two longitudinal studies [71,72]. On the other hand, PA equipment was inversely associated with sedentary behaviours in three of six studies [55,75,77], although no longitudinal studies investigated this relationship. The least investigated category of the home physical environment was the house and yard. Two of six cross-sectional studies found that yard space was positively associated with a PA measure [58,68] and one of these studies also found living in an apartment was negatively associated [58]. No studies investigating sedentary behaviours included a measure of size, space or design of the house and yard.

### Relationships between home physical and social environmental factors

The majority of observational studies investigating the home physical environment also investigated home social environmental variables. Measures included family rules (19 studies), family social support (14 studies) and family behaviours (10 studies). Significant relationships between the home social environment and sedentary behaviours, and to a lesser extent PA, were evident after adjusting for home physical environmental factors. Some form of family social support was positively associated with sedentary behaviours in six of seven cross-sectional studies [41,43-45,49,76]. For PA outcomes five of eight studies showed an association in the expected direction. Social support for PA was positively associated with PA in four studies, including two longitudinal studies [62,64,71,72], and playing e-games with parents was positively associated with low activity in one study [76]. Four of these studies scored the highest internal and external validity [64,71,72,76]. Six of eight studies on electronic media use, with a family behaviour measure, found parental electronic media use positively associated with children’s use [44,46,47,52,56,76]. This included all studies investigating TV time. Of the three studies measuring parental behaviour and PA outcomes, one cross-sectional study found parent e-game use positively associated with low activity [76], and one longitudinal study found parent PA participation positively associated with MVPA [71]. Both studies scored the highest internal and external validity. Of the six studies investigating rules and PA, these same two studies found an association. The longitudinal study found a positive relationship between rules restricting PA and MVPA in girls [71], and the cross-sectional study found mixed results with supervision of TV positively associated with low activity in boys and negatively associated in girls [76]. Electronic media rules were negatively associated with sedentary behaviours in 11 of 14 studies, including the one relevant longitudinal study [43,45,46,49-52,54-56,76].

In the 15 studies with sedentary outcomes that measured both physical and social environmental variables, 10 found associations with physical and social environmental measures [41,43-46,50,51,54-56]. Of these, only one study investigated an interaction between the home physical and social environmental factors reviewed, and results showed an inverse association between parental rules and TV viewing only when there was a TV in the bedroom [52]. In the 10 studies with PA outcomes that investigated the home physical and social environment, one study found associations with both [76]. No other moderating or mediating relationships between the reviewed physical and social environmental factors were explored.

### Experimental studies

Experimental studies used one of two strategies to change the home physical environment: either introducing a television limiting device or an active video game (AVG).

#### Television limiting device studies

Five randomised control trials (RCT), ranging in duration from six weeks to 12 months, introduced a television limiting device (Table 4). Four studies scored the highest internal validity [79-81,89], while no studies scored the highest external validity. All studies measured screen based sedentary outcomes [79-81,85,89], and four also measured PA and body composition outcomes [79-81,89]. Three studies of the highest internal validity found a significant decrease in TV viewing in the intervention group (47, 73 and 116 minutes per day) [80,81,89]. Two of these also showed improvement in body mass index (BMI) [81,89]. The one study that rewarded children for PA with TV viewing tokens also increased PA by 65% [89]. Of the two studies that did not significantly change children’s sedentary behaviours, one found a significant decrease in overall household TV watching [79].

**Table 4:** Summary of experimental studies including TV limiting devices

**Author (Year); Country**	**Sample characteristics (Number; Age; Sex; Other)**	**Intervention description (Design; Duration; Characteristics)**	**Measures**	**Summary of key findings**	**Adjustments**	**Internal validity**	**External validity**
French (2011); USA [79]	n=75 adolescents randomised, n=87 HHs; 12–17 yrs; Sex not reported; HH TV ≥ 10 hrs per person per wk.	Cluster RCT; 12 mths; Intervention - TV limiting devices, guidelines about food availability, 6 x group sessions, behavioural strategies, phone calls, 12 x home-based activities; Control - no intervention.	SB - TV (SR); PA - MVPA (SR); Other - zBMI, dietary intake, eating behaviours, PA encouragement, PA with others in HH, TV is on.	Significant decrease in reporting TV is on, and significant increase in consumption of fruit and veg in intervention compared to control. At HH level there was a significant decrease in TV watching, and a significant increase in PA encouragement, PA with others in HH compared to control.	Gender, smoking, age, HH income, configuration, race, education; baseline values of outcomes; HH clustering.	++	+
Goldfield (2006); Canada [89]	n=30; 8–12 yrs; 43% M; overweight or obese, TV/video games ≥15 hrs per wk, <30 mins MVPA per day.	RCT; 8 wks; Intervention - wore PA monitor (open-loop feedback) and rewarded for PA (reinforcement) with TV access via token controlled TV limiting device; Control - wore a PA monitor (open-loop feedback only).	SB - TV based, other (SR); PA - activity counts, MVPA, VPA (Acc); Other - height, weight, BMI, dietary intake.	Significantly greater changes in total activity counts and MVPA, and reduction in TV based SB, fat intake, calories from snacks and snack intake in front of TV, and improvement in weight and BMI, compared to control. Reductions in weight, fat intake, calories from snacks, calories consumed in front of TV significantly correlated with reduction in TV based SB.	Not noted.	++	-
Ni Mhurchu (2009); New Zealand [85]	n=29; 9–12 yrs; 62% M; TV > 20 hrs per wk.	RCT (Pilot); 6 wks; Intervention - electronic TV monitors, encouraged to restrict TV to 60 mins per day, ideas to reduce TV; Control - ideas to reduce TV.	SB - TV, total screen time (SR); PA - steps (pedometer); Other - BMI, energy intake from snacks; Interviews.	No significant differences. Decrease in weekly TV of 254 mins in intervention and 3 mins in control (NS). Total screen time decreased and steps increased slightly in both groups (NS). Mixed views on family acceptability of TV time monitors.	Baseline values of outcomes.	+	+
Robinson (2006); USA [80]	n=181; mean 8.9 yrs; 54% M.	Cluster RCT; 6 mths; Intervention - SMARTschool curriculum (18 lessons with TV Turn Off Challenge and goal to reduce to 7 hrs per wk), TV allowance device, parent newsletters; Control - no intervention.	SB - TV, video, video game play (SR); Other - family member TV viewing; Interviews.	Significant reduction in weekday TV, and weekday and weekend video game play compared to control. Significant reduction in mother, father and sibling TV viewing compared to control. Age, supervision, and prior TV and video game use moderated intervention effects.	Baseline values of outcomes.	++	+
Todd (2008); USA [81]	n=21; 8–11 yrs; M only; TV > 3.5 hrs or EM > 5.8 hrs per day.	RCT; 20 wks; Intervention - seminar including goal setting, newsletters, TV allowance device, software to limit computer use, phone calls, recommendation to reduce EM to 90 mins per day; Control - no intervention.	SB – EM use (SR); PA - steps (pedometer); Other - height, weight, BMI, % body fat, snacks and meals consumed with EM, dietary intake, bone mineral density.	Significant treatment by time interaction for EM use and % body fat. Intervention decreased EM use from 153mins per day to 81 (10 wks) and 82 (20 wks) and control from 157 to 119 and 95 (adjusted difference of 73 mins at 20 weeks); Intervention decreased % body fat from 26.1 to 24.6 (20 wks) and control increased from 27.7 to 28.0. Significant reduction in snacks and meals consumed with EM, compared to control.	Organised activity, electronic media access.	++	-

*M* male, *HH* household, *SB* sedentary behaviour, *PA* physical activity, *RCT* randomised control trial, *MVPA* moderate to vigorous physical activity, *NS* non-significant; n= is number in analysis unless noted; *BMI* body mass index, *EM* electronic media, *Acc* accelerometer, *SR* self reported; Measures column includes outcomes and variables used in further analysis (excluding adjustment variables); Significance at p<0.05 (outcomes not reported or listed in table as non-significant if p > 0.05); Adjustments listed as stated in paper.

#### Active video gaming studies

Of the six experimental studies that introduced an AVG into the home, four were RCTs (Table 5). Two RCTs scored the highest internal validity [84,86], while no studies scored the highest external validity. Study durations ranged between 12 weeks and six months. Five of six studies collected outcome measures mid-intervention. Of the three RCTs that compared an intervention group to a play as usual control group: one found an increase in AVG play of 57 minutes per day at mid-intervention [84]; one found an increase in AVG play of 10 minutes per day and improvement in BMI post-intervention [86]; and one found an average difference in sedentary video gaming of 52 minutes per day, an increase in PA at mid-intervention and improvement in waist circumference post-intervention [87]; Two studies found a significant decrease in AVG play between the first and second half of the intervention [83,84], with another two showing non-significant decreases [82,88].

**Table 5:** Summary of experimental studies including active video games

**Author (Year); Country**	**Sample characteristics (Number; Age; Sex; Other)**	**Intervention description (Design; Duration; Characteristics)**	**Measures**	**Summary of key findings**	**Adjustments**	**Internal validity**	**External validity**
Chin A Paw (2008); The Netherlands [88]	n=16; 9–12 yrs; 14% M; low fitness.	RCT (Pilot); 12 wks; Multiplayer intervention - Interactive Dance Simulation Video Game (IDSVG) for home use, 60 min weekly group class; Home intervention - IDSVG for home use only.	PA - ISDVG play (SR); Other - focus groups.	Multiplayer group averaged 901 mins ISDVG play and home group 376 mins (NS); Median play decreased from 228 mins in first 6 weeks to 0 min in second 6 weeks for home group, and increased from 475 min to 601 min in multiplayer group (NS). Significantly lower drop out in multiplayer group (15%) compared to home group (64%); Technical difficulties, need for computer and space, dull music and becoming bored were barriers.	Not noted.	+	-
Graves (2010); England [84]	n=42; 8–10 yrs; %M not reported.	RCT; 12 wks; Intervention - video games linked to jOG device that required stepping; Control - video game play as usual.	PA - Step powered video gaming, AVG play, total video gaming (SR), steps, CPM, total PA (Acc); SB - sedentary video gaming, TV, productive behaviours, leisure behaviours (SR), sedentary (Acc); Other - stature, body mass, BMI, maturity offset, subtotal body fat, trunk body fat.	Significant increase in AVG play compared to control at 6 weeks; Step powered video gaming was significantly higher at week 6 than 12 in intervention group.	Gender; baseline values of outcomes; change in maturity offset (some).	++	+
Maddison (2011); New Zealand [86]	n=322; 10–14 yrs; 73% M; overweight/ obese, video games ≥ 2 hrs per wk.	RCT; 24 wks; Intervention - AVG supplied, encouraged to do 60 mins PA per day; Control - video game play as usual.	PA - AVG play (SR), MVPA (Acc); SB - sedentary video gaming (SR); Other - weight, BMI, zBMI, total body fat, % body fat, waist circumference, energy intake from snacks, fitness.	Significant treatment effect on zBMI, BMI, % body fat, total body fat, and increase in active video game time compared to control.	Age, sex, ethnicity; baseline values of outcomes.	++	+
Madsen (2007); USA [82]	n=30^A^; 9–18 yrs; 40% M; obese.	Pre/post design; 6 mths; Intervention - DDR game, instructed to use 30 min x 5 days a wk, biweekly phone calls; No control group.	PA – Dance Dance Revolution (DDR) use (SR), energy expenditure (memory card); Other - BMI; Interviews.	No significant effects. 12 children used DDR at least twice a week in first 3 months, and only 2 in second 3 months. Family stressors and boredom were barriers.	Baseline zBMI.	-	-
Ni Mhurchu (2008); New Zealand [87]	n=20; 10–14 yrs; 40% M.	RCT (Pilot Study); 12 wks; Intervention - AVG supplied, instructed to substitute for regular video games; Control - video game play as usual.	PA - AVG time, total video gaming (SR), CPM (Acc), MVPA (SR); SB - inactive video gaming (SR); Other - BMI, waist circumference.	Average time in inactive video gaming was significantly lower compared to control. Objective PA (CPM) (6 wks) significantly higher and waist circumference (12 wks) significantly improved compared to control. Average total video game time was lower (54 vs 98 mins per day) compared to control (NS).	Sex; baseline values of outcomes.	+	+
Owens (2011); USA [83]	n=12 children, n=8 families; 8–13 yrs; 50% M.	Pre/post design; 3 mths; Intervention - Wii Fit, no instruction; No control group.	PA - PA (Acc), Wii Fit use (console memory); Other - height, weight, %body fat, BMI, balance, muscular fitness, aerobic fitness, flexibility.	12 min average Wii Fit use per HH per day, which decreased significantly from first to second 6 wks (21.5 to 3.9 mins per day). No significant pre-post changes in children except height and V02.	Not noted.	+	-

*M* male, *HH* household, *SB* sedentary behaviours, *PA* physical activity, *RCT* randomised control trial, *MVPA* moderate to vigorous physical activity, *NS* non-significant; n= is number in analysis unless noted; *BMI* body mass index, *Acc* accelerometer, *SR* self reported, *CPM* counts per minute; Measures column includes outcomes and variables used in further analysis (excluding adjustment variables); Significance at p<0.05 (outcomes not reported or listed in table as non-significant if p > 0.05); Adjustments listed as stated in paper; *A* n =26 at 3 mths, 21 at 6 mths, 12 with DDR use diary, 7 had memory card.

## Discussion

The purpose of this paper was to review the influence of the home physical environment on children’s PA and sedentary behaviour. Results showed that media equipment was positively associated with screen based sedentary behaviours and PA equipment was unrelated to PA, reinforcing results of earlier reviews. Several previously unreviewed relationships were summarised, highlighting an inverse relationship between PA equipment and sedentary behaviour in half of studies. Interventions that changed the home environment by introducing TV limiting devices reduced TV time. The social environment, in particular parents, played an important role in influencing children’s sedentary behaviour and PA even in the presence of home physical environmental factors. The field is limited by the lack of objective assessment and no investigation of the indoor home space beyond equipment. Additionally, there was a paucity of studies investigating objectively measured sedentary time and home context specific behaviours. This review extends previous knowledge by critically assessing and synthesising evidence from both experimental and observational studies. The paper identifies current evidence gaps and measurement issues, and generates future directions for research on children’s sedentary behaviour and PA within the home space.

### Research evidence and gaps

#### The home - house and yard

In the current review, investigation of the size, space and design of the house and yard was limited. Although, previous reviews of correlates of children’s physical activity have not addressed this relationship at all [14,15,28,29]. Only six studies could be found that collected any measure of the house or yard and the presence of a garden was the only measure collected more than once. While one of three studies investigating outdoor play found that girls without a garden played outside less [58], our overall results are in contrast to the findings of several qualitative studies that have identified lack of yard space as a barrier to physical activity and active play [23,24,26]. This lack of association could have been influenced by the limited and categorical nature of environmental variables investigated, and outcome measures that were not specific to the home. With the majority of children’s MVPA occurring outside of the home [90,91], and the majority of leisure time at home spent indoors and sedentary [33,34], it would seem pertinent to investigate the influence of the house and yard on children’s sedentary behaviour independent of PA. Further investigation of the indoor home space may also be relevant for PA with a recent ecological momentary assessment study of 9–13 year olds in California finding that 30% of all leisure time PA occurred indoors at home and only 8% occurred in the yard at home [90]. To date there has been no exploration of the relationship between the house and yard and children’s sedentary behaviours or home context specific PA.

#### The home – media equipment

Observational studies showed a positive relationship between media equipment and children’s screen based sedentary behaviours. This extends the findings of earlier reviews that have located few or no studies investigating this relationship [13,14] and concurs with a more recent review of sedentary behaviour correlates [16]. The review also considered media equipment and PA, a relationship not summarised by previous reviews [14,15,28,29], and found the majority of studies showed no relationship.

This review located three studies investigating media equipment and objectively measured sedentary time and, to our knowledge, is the first review to summarise this relationship. Findings on the influence of media equipment in the home were mixed and there was no association between bedroom media equipment and accelerometer measured sedentary time [53,73,77]. Even with limited evidence, these findings are curious given the associations between media equipment and screen based sedentary behaviour. This suggests that children with less media equipment at home may simply substitute one sedentary behaviour for another resulting in no discernible difference to overall sedentary time. This is consistent with qualitative findings that indicated children would consider both active and sedentary alternatives if screen viewing was limited [25]. Notably, the studies reviewed measured sedentary time across the entire day. This included sedentary time in school and other places outside the home, which may be less likely to be influenced by home media equipment.

#### The home – physical activity equipment

Overall, our results support previous conclusions of limited evidence for a relationship between PA equipment at home and children’s PA [14,28,29]. However, we also found that children with more PA equipment spent less time in sedentary behaviours in half of studies [55,75,77], a relationship not considered by previous reviews [13,14,16]. While this evidence is inconclusive, it does raise the possibility that PA equipment at home may decrease sedentary behaviours by prompting alternative light intensity activities rather than MVPA.

#### Changing the home environment

Changing the home physical environment has the potential to influence children’s sedentary behaviour and PA. This review found interventions that implemented a TV limiting device were successful in decreasing children’s screen based sedentary behaviour. Also, introducing an AVG to the home resulted in positive changes in AVG play, sedentary electronic game play, PA and/or body composition in some studies [84,86,87], although early changes did not always last. Interventions that showed the most promise in changing behaviour and/or body composition directed participants to substitute activities, such as active gaming for sedentary gaming, or to earn TV time by participating in PA [84,87,89]. However, the acceptability of these changes in the home was debatable, with the need for space, boredom and disruption of other family members cited [82,85,88]. Additionally, the long term effects of introducing an AVG or limiting TV viewing on other behaviours, including screen based sedentary behaviours, productive sedentary behaviours and PA, are unclear.

#### Home physical and social environmental interactions

The review reaffirms the important role that parents play in supporting, restricting and normalising children’s sedentary behaviours [13,16]. In accordance with ecological frameworks, we found that the influence of social environmental factors in the presence of physical environmental factors was evident. Although, it should be noted that results relating to the home social environment are limited to studies that investigated both social and physical environmental factors. Children with parents who watched more TV and those in families with no electronic media rules, spent more time watching TV. Additionally, children who participated in sedentary behaviour with their parents spent more time in sedentary behaviour. Despite many studies based upon ecological models, few investigated relationships between physical and social environmental factors within the home space. Of those that did, one study found parental rules were only effective when there was a bedroom TV [52]. Parents largely dictate the arrangement of the home space and determine the equipment available to children at home. Family rules are of particular interest as they present an avenue for controlling the influence of the physical environment through not allowing screens in bedrooms and living areas or choosing not to purchase media equipment. They may also restrict or encourage the use of home space for active behaviours. Thus far few studies have explored these relationships.

### Measurement issues

Other authors have called for behaviour specific measures of the environment and context specific measures of PA and sedentary behaviours in the investigation of environmental influences [19,92,93]. Our review affirms the need for increased specificity of research on the home environment. There was only one study with a home context specific PA outcome [69]. Also, while specific sedentary behaviours such as TV watching mostly occur at home, objectively measured sedentary time was accumulated across the entire day. Reinforcing the value of this approach, we found elements of the home physical environment were more consistently associated with sedentary behaviours, which are more likely to occur at home, than with PA outcomes.

Studies of the neighbourhood built environment have used objective measures (audits and Geographic Information Systems (GIS)) as well as perceived measures (self-report) [22,94] to assess the environment, and Global Positioning Systems (GPS) to track individuals’ movement [95]. This review found all but one study used surveys to measure the home environment, with the exception using an inventory that measured equipment density, availability and accessibility [77]. Supporting the case for more robust measurement, this study was the only one to find PA equipment, in this case equipment density, related to accelerometer measured MVPA. It also found the ratio of PA to media equipment was related to accelerometer measured sedentary time. While it is acknowledged that current GIS and GPS technologies provide limited utility indoors, other technologies combining indoor location and movement sensors may have potential, but are yet to be applied in this context [96].

Technology available within homes is changing rapidly and this also has implications for measurement of home media. For example electronic gaming may now be active or sedentary and portable media devices can be used in different places within the home [97]. However, in this review, only two observational studies included these more recent leisure technologies [75,77].

### Future research directions

Future studies on the influence of the home physical environment on children’s sedentary behaviour and PA need to investigate home context specific outcomes. The investigation of objectively measured sedentary time at home is most important [98]. Sedentary time at home should also be categorised by the type of sedentary behaviour including screen based and other productive/non-productive behaviours, such as reading and homework. This will assist to clarify whether children who spend less time in screen based behaviours are overall less sedentary at home, or whether they do more of other non-screen sedentary behaviours [33]. For PA, time spent in objectively measured PA levels at home and activity behaviours that occur specifically at home such as active play, AVG play and household chores are most relevant for future research.

Investigation of the home physical environment lacks objective assessment and is limited, except for equipment. Future research on the indoor and outdoor home environment should, where applicable, adopt approaches used to measure the outdoor built environment. For example, GIS can provide objective measures of house and yard size [99] and audits provide scope to collect more objective and detailed data inside the home [77]. Additionally, just as GPS has been applied to locate individuals in outdoor environments [95], newer measurement technologies, such as indoor location and movement sensors, provide an avenue to track the location of individuals in indoor space [96]. Future research should draw on established approaches from other fields and closely monitor the development of new technologies that have measurement potential within the home.

Both social and physical environmental factors influence children’s sedentary behaviour and PA, yet how they interact within the home space remains unclear. Parents control many elements of the home physical environment. Future research should explore the relationships between parental behaviour, family rules, equipment and arrangement of the home space, to better understand how sedentary behaviour is influenced by the home environment and to inform the development of interventions.

Further intervention studies are highly recommended. We found only two types of interventions that changed the home physical environment. TV limiting devices reduced TV viewing, however, it is unknown which behaviours replace TV viewing and whether this strategy is successful in the longer term. Similarly, introducing an AVG showed promise, but any effects seemed to decay quickly. Interventions are required to determine the longer term effects of introducing TV limiting devices and AVGs on activity levels, and any unintended consequences on other physical activities and sedentary gaming. Other strategies such as changing the location of media equipment within the home, reconfiguring indoor spaces and substituting sedentary behaviours with active alternatives are lacking and should be explored. The key challenge for future research is to find acceptable active alternatives to traditionally sedentary behaviours within the home space.

### Strengths and limitations of the review

This review included the best available evidence from both observational and intervention studies, identifying a larger number of studies with relevant home physical environmental variables than previous reviews. However, there are some limitations to the process and scope of this review. Firstly, some studies may have been missed due to the nature of the search terms and there may be some publication bias to studies with significant results. Secondly, independent and outcome measures were pooled into categories which were useful for summarising evidence, but did not differentiate between very specific environmental measures. For example, the home media equipment category included presence of a TV, presence of a computer, density of media equipment and number of TVs in the home. Thirdly, the summary of the home social environment in isolation should be interpreted with caution as it was limited to papers that also included home physical environmental factors and only the most common social home environmental factors were investigated. Also, individual factors were not included in the review. Finally, the pre-adolescent age group was identified as particularly relevant for investigation, although we acknowledge that this age group encompasses both children and adolescents as defined in previous reviews, and may limit comparability.

## Conclusion

This review found that both physical and social environmental factors operating within the home space are important influences on children’s sedentary behaviour and PA. Media equipment is associated with children’s screen based sedentary behaviours. Changing the physical environment shows promise for reducing the sedentary nature of homes, although further interventions are needed to understand the broader impact of changes. Considering the substantial amount of time children spend at home, there has been little investigation of how the physical parameters of the home space may constrain or support children’s sedentary behaviour and PA. Future studies should ideally include objective measures of the home and prioritise investigating environmental influences within the home space on objectively measured sedentary time at home and home context specific behaviours.

## Competing interests

The authors declare that they have no competing interests.

## Authors’ contributions

CM contributed to the conceptualisation and design of the manuscript, conducted the literature search, quality assessment of papers and analysis, and drafted the manuscript. GS, SF, RB, & MR contributed to the conceptualisation of the manuscript and verification of papers, and provided critical feedback on the manuscript. All authors read and approved the final manuscript.
